# Crystal structure, characterization and Hirshfeld analysis of bis­{(*E*)-1-[(2,4,6-tri­bromo­phen­yl)diazen­yl]naphthalen-2-olato}copper(II) dimethyl sulfoxide monosolvate

**DOI:** 10.1107/S2056989020001863

**Published:** 2020-02-18

**Authors:** Souheyla Chetioui, Hassiba Bougueria, Ouarda Brihi, Mehdi Boutebdja, Nadia Bouroumane, Hocine Merazig, Rachid Touzani

**Affiliations:** a Unité de Recherche de Chimie de l’Environnement et Moléculaire Structurale (CHEMS), Faculté des Sciences Exactes, Département de Chimie, Université des Frères Mentouri Constantine, Algeria; b Faculté de Technologie, Université Mohamed Boudiaf M’sila, Algeria; c Université abd el Hafid Boussouf, Mila, 43000 Mila, Algeria; d Laboratoire de Cristallographie, Département de Physique, Université Mentouri-Constantine, 25000 Constantine, Algeria; e Ecole Nationale Polytechnique de Constantine, Constantine, Algeria; f Laboratoire de Chimie Appliquée et Environnement (LCAE), Département de Chimie, Faculté des Sciences, Université Mohamed Premier, BP 524, 60000, Oujda, Morocco; g Laboratoire de Chimie, Faculté des Sciences, Département de Chimie Appliquée et Environnement, Université Mohammed Premier, Oujda, Morocco

**Keywords:** Crystal structure, Azo dyes, Cu^II^ complex, (N,*O*)-ligands., crystal structure

## Abstract

In the title compound, [Cu(C_16_H_8_Br_3_N_2_O)_2_]·C_2_H_6_OS, the Cu^II^ atom is tetra­coordinated in a square-planar coordination, being surrounded by two N atoms and two O atoms from two *N*,*O*-bidentate (*E*)-1-[(2,4,6-tri­bromo­phen­yl)diazen­yl]naphthalen-2-olate ligands. The two N atoms and two O atoms around the metal center are *trans* to each other, with an O—Cu—O bond angle of 177.90 (16)° and a N—Cu—N bond angle of 177.8 (2)°. The average distances between the Cu^II^ atom and the coordinated O and N atoms are 1.892 (4) and 1.976 (4) Å, respectively. In the crystal, complexes are linked by C—H⋯O hydrogen bonds and by π–π inter­actions involving adjacent naphthalene ring systems [centroid–centroid distance = 3.679 (4) Å]. The disordered DMSO mol­ecules inter­act weakly with the complex mol­ecules, being positioned in the voids left by the packing arrangement of the square-planar complexes. The DMSO solvent mol­ecule is disordered over two positions with occupancies of 0.70 and 0.30.

## Abstract 2

In the title Cu^II^ complex, a newly synthesized dye, the metal atom is coordinated by two N atoms and two O atoms from two bidentate (*E*)-1-[(2,4,6-tri­bromo­phen­yl)diazen­yl]naphthalen-2-olate ligands.

## Chemical context   

Azo dyes are an important class of organic compounds that are attractive to researchers because of their various applications (Zollinger, 1961 ▸; Nishihara, 2004 ▸; Sahoo *et al.*, 2015 ▸). They constitute the largest group of azo compounds and are the most widely used colorants in the industry. Applications of azo dyes include their use as coloring agents because of their affinity for wool and silk (Patel *et al.*, 2011 ▸), in photoelectronics (Sekar, 1999 ▸), optical storage technology (Wang *et al.*, 2000 ▸), biological reactions (Węglarz-Tomczak *et al.*, 2012 ▸), printing systems (Abe *et al.*, 1999 ▸; Dharmalingam *et al.*, 2011 ▸), in analytical areas (Abdalla *et al.*, 2013 ▸; Amin *et al.*, 2003 ▸) and in the food industry (Almeida *et al.*, 2010 ▸). Azo derivatives and their metal complexes are important homologue pigments for synthetic leather and vinyl polymers. Furthermore, azo compounds are known to be involved in a number of bio­logical reactions, such as inhibition of DNA, RNA, and protein synthesis, nitro­gen fixation and carcinogenesis (Badea *et al.*, 2004 ▸). In addition, high-density optical data storage has been the subject of extensive research over the past decade. In general, cyanine dyes, phthalocyanine dyes, and metal-azo dyes are used in the recording layer of DVD-R (digital versatile disc-recordable) discs. It was reported that the new technology, which employs 405 nm blue–violet diode lasers, will require a new optical-recording medium matching the 405 nm wavelength laser (Steed *et al.*, 2007 ▸). In comparison with the dyes themselves, metal-azo dyes are light-stable, allow an easier control of the wavelength by selection of the appropriate substituent groups, and have good thermal stability (Geng *et al.*, 2004 ▸; Bin *et al.*, 2003 ▸; Fu-Xin *et al.*, 2003 ▸; Hamada *et al.*, 1997 ▸; Suzuki *et al.*, 1999 ▸; Nejati *et al.*, 2009 ▸; Li *et al.*, 2010 ▸). Being inter­ested in the synthesis and preparation of metal complexes bearing such ligands, we have synthesized and structurally characterized Cu^II^ complexes with *N,O-*bidentate phenyl­azo-naphtho­late ligands (Chetioui *et al.*, 2015*a* ▸,*b* ▸). In our previous work, we were inter­ested by the colour-generation mechanism of azo pigments, usually characterized by the chromophore of the azo group (–N=N–) (Bougueria *et al.*, 2013*a* ▸,*b* ▸,*c* ▸, 2014 ▸; Chetioui *et al.*, 2013*a* ▸,*b* ▸). Herein, we report the synthesis and crystal structure of a Cu^II^ complex incorporating the ligand (*E*)-1-[(2,4,6-tri­bromo­phen­yl)diazen­yl]naphthalen-2-ol, for which the structure is known (Chetioui *et al.*, 2013*a* ▸).
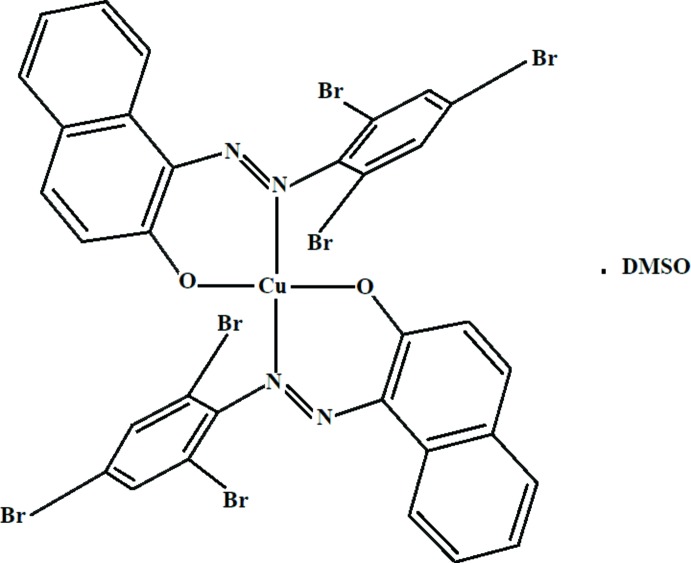


## Structural commentary   

**Figure 1:**
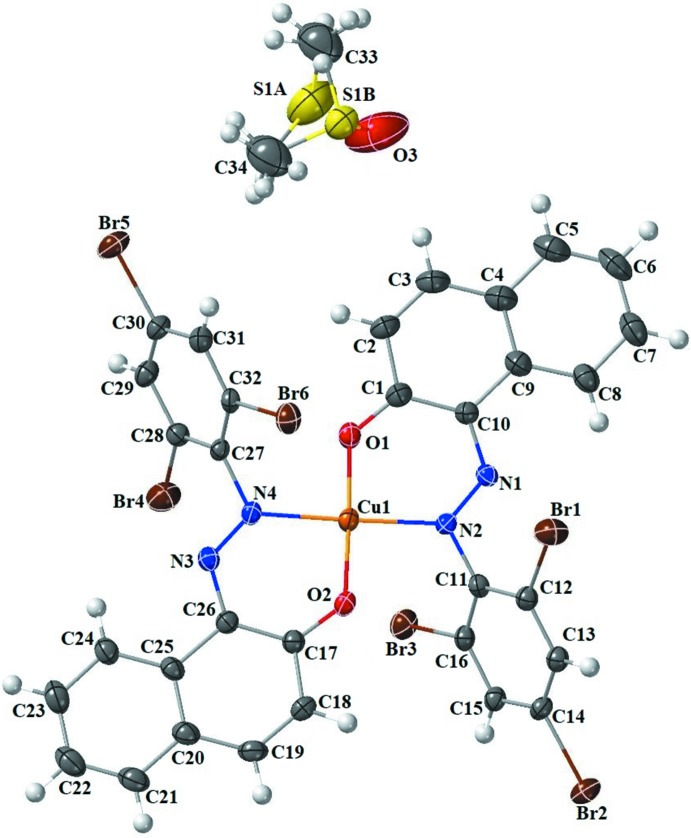
The mol­ecular structure of the title compound with atom labelling and displacement ellipsoids drawn at the 50% probability level.

The structure of the title compound is shown in Fig. 1 ▸. The asymmetric unit consists of a Cu^II^ complex mol­ecule and a DMSO solvent mol­ecule. In the complex, the Cu^II^ atom is coordinated by two oxygen and two nitro­gen atoms *trans* to each other. The Cu1—N2 and Cu1—N4 bond lengths [1.976 (4) and 1.971 (5) Å, respectively] are almost identical. The N—Cu—N bond angle is 177.8 (2)°. The two Cu—O distances are 1.882 (4) and 1.892 (4) Å. All bond lengths are similar to those observed in similar crystal structures (Chetioui *et al.*, 2015*a* ▸,*b* ▸). The N—Cu—O bond angles range from 88.75 (18) to 93.06 (17)° and the O—Cu—O angle is 177.90 (16)°. Therefore, the copper atom can be considered to be in a slightly distorted square-planar geometry. The dihedral angle formed between the plane of the C1–C10 naphthalene ring system and the tri­bromo­benzene ring is 51.4 (2)°.

## Supra­molecular features   

**Figure 2:**
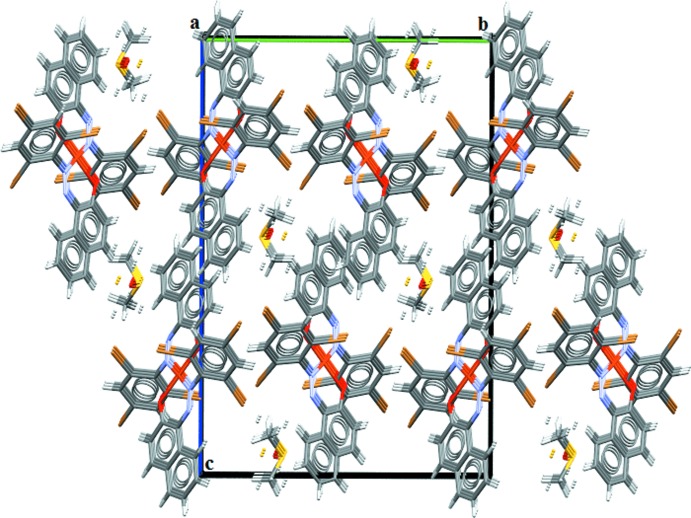
A view along the *a* axis of the crystal packing of the title compound.

In the crystal, the complex mol­ecules and the DMSO mol­ecules are linked by C3—H3⋯O3 and C23—H23⋯O3 hydrogen bonds (Table 1 ▸), forming parallel complex–solvate chains along the *b*-axis direction (see Fig. 2 ▸). π–π stacking inter­actions involving adjacent naphthalene ring systems [centroid–centroid distance = 3.679 (4) Å] are observed between complex mol­ecules.

## Analysis of the Hirshfeld surfaces   

**Figure 3:**
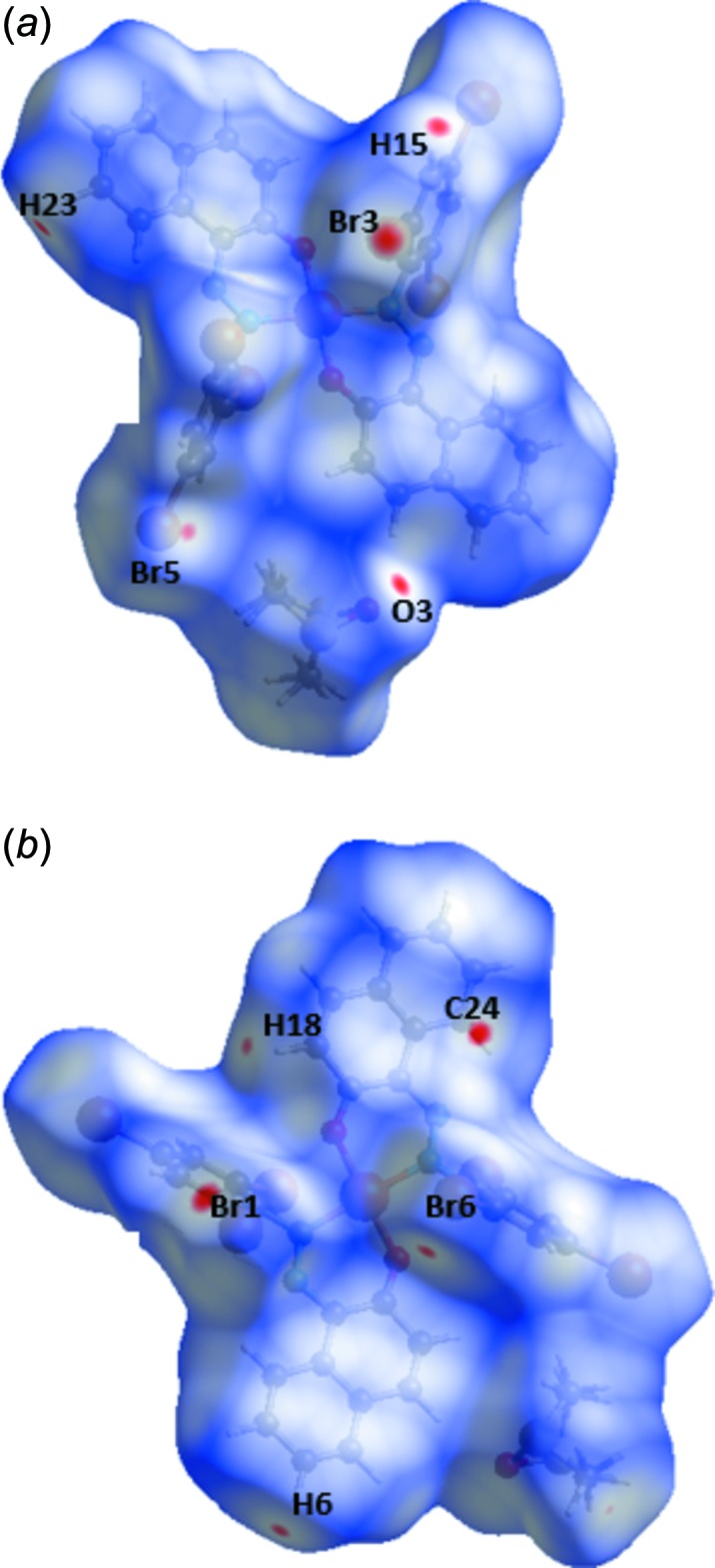
View of the Hirshfeld surface mapped over *d*_norm_.

**Figure 4:**
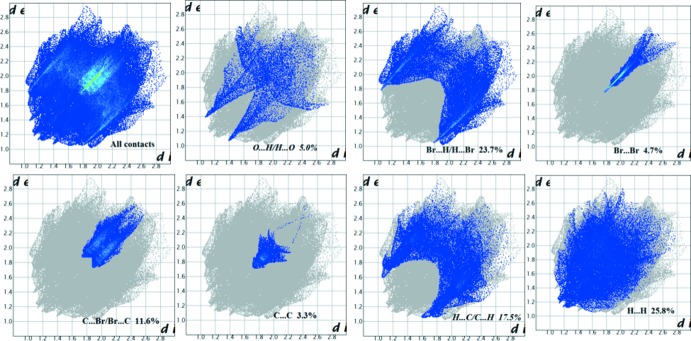
Two-dimensional fingerprint plots of the compound showing (*a*) all inter­actions and those delineated into (*b*) H⋯O/O⋯H, (*c*) Br⋯H/H⋯Br, (*d*) Br⋯Br, (*e*) C⋯Br/Br⋯C, (*f*) C⋯C, (*g*) H⋯C/C⋯H and (*h*) H⋯H inter­actions.

**Figure 5:**
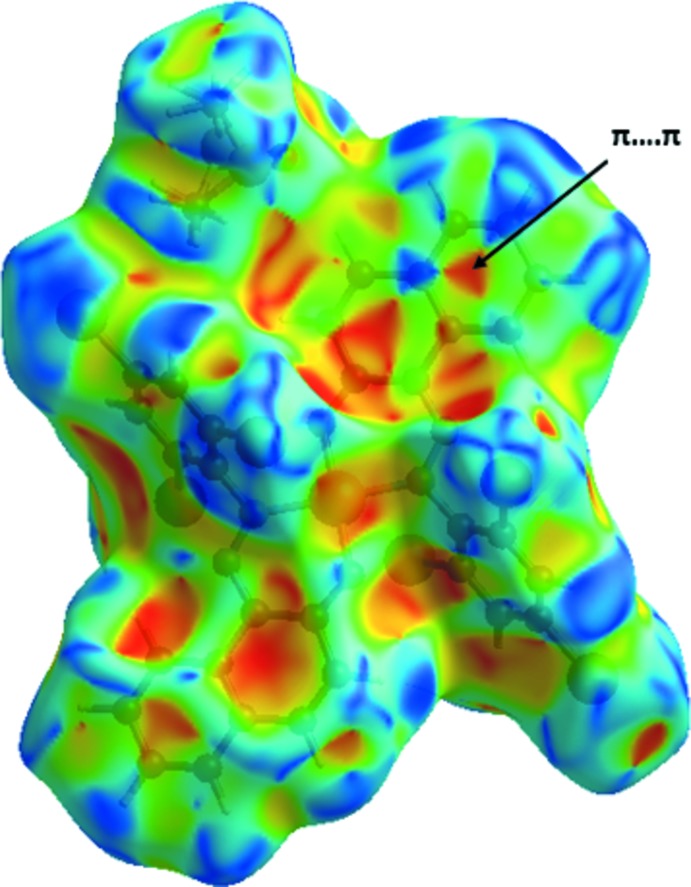
Hirshfeld surface mapped over shape-index, highlighting the region involved in π–π stacking inter­actions.

The program *Crystal Explorer 3.1* (Wolff *et al.*, 2012 ▸) was used to generate the Hirshfeld surface (Spackman & Jayatilaka, 2009 ▸) mapped over *d*_norm_ (Fig. 3 ▸). The bright-red spots correspond to the H⋯·O/O⋯H close contacts (C—H⋯O hydrogen bonds), while the faint-red spots, near the H⋯O contacts, are attributed to Br⋯H, Br⋯Br and C⋯H contacts. The white areas correspond to regions where the distances separating neighboring atoms are close or equal to the sum of the van der Waals radius of the atoms. The corresponding fingerprint plots (McKinnon *et al.*, 2007 ▸) are shown in Fig. 4 ▸. The relative contributions from the different inter­atomic contacts to the Hirshfeld surfaces are as follows: O⋯H/H⋯O contacts 5.0%, H⋯Br/Br⋯H 23.7%, Br⋯Br 4.7%, Br⋯C/C⋯Br 11.6%, C⋯C 3.3%, C⋯H/H⋯C 17.5%, H⋯H 25.8%. The presence of π–π stacking inter­actions are indicated in the Hirshfeld surface mapped over shape-index (Fig. 5 ▸).

## Synthesis and crystallization   

The complex, bis-1-(2,4,6-tri­bromo­phenyl­azo)-2-naphtho­latecopper(II), was obtained by mixing 1 mmol of 1-(2,4,6-tri­bromo­phenyl­azo)-2-naphthol dissolved in 20 ml of THF with 0.5 mmol of Cu(OAc)_2_·H_2_O dissolved in 20 ml of MeOH. The mixture was refluxed at 333 K for 8 h. Upon cooling, a dark-orange solid was observed, which was filtered off and washed with water, and then dried under vacuum. Crystallization in DMSO yielded 83% of a crystalline material. To confirm the formula of the solvate complex, an elementary analysis was carried out: calculated for C_32_H_16_Br_6_CuN_4_O_2_·C_2_H_6_OS, C 36.80%, N 5.05%, H 2.00%, found C 36,27%, N 4,81%, H 1,92%. The ^1^H NMR spectrum (paramagnetic complex) shows a multiplet around 7 and 8 ppm attributed to the aromatic protons. The IR spectrum of the complex shows the vibration bands: ν(N=N); 1360 cm^−1^, ν(C—N): 1149 cm ^−1^, ν(C—Br): 645 cm^−1^, ν(C—O): 1207 cm^−1^ (aromatic), ν(C=C): 1498 cm^−1^ (aromatic), ν(C—H): 2945 cm^−1^ (aromatic), ν(Cu—N): 417 cm^−1^, ν(Cu-O): 558 cm^−1^. The UV–Vis spectrum measured in CH_2_Cl_2_ (10 ^−5^ M), shows three absorption bands: an intense band at 268 nm (∊ = 29.94 10^8^ M^−1^ cm^−1^) attributed to intra-ligand charge-transfer transition, a band at 382 nm (∊ = 79.21 10^7^ M^−1^ cm^−1^) associated with the azo form of the ligand and a band at 462 nm (∊ = 63.84 10^7^ M^−1^ cm^−1^) attributed to metal–ligand charge transfer.

## Refinement   

Crystal data, data collection and structure refinement details are summarized in Table 2 ▸. The H atoms were included in calculated positions and treated as riding atoms: C—H = 0.93 Å with *U*_iso_(H) = 1.2 *U*_eq_(C). An absorption correction was not applied in view of the very small size of the crystal [0.1 × 0.09 × 0.08 mm]. The DMSO solvent mol­ecule shows disorder over two positions with final occupancies of 0.70 and 0.30. The disordered atoms were modelled as anisotropic using EADP restraints. H atoms of the disordered DMSO were omitted.

## Supplementary Material

Crystal structure: contains datablock(s) global, I. DOI: 10.1107/S2056989020001863/tx2018sup1.cif

Structure factors: contains datablock(s) I. DOI: 10.1107/S2056989020001863/tx2018Isup2.hkl

CCDC reference: 1983017

Additional supporting information:  crystallographic information; 3D view; checkCIF report

## Figures and Tables

**Table 1:** Hydrogen-bond geometry (Å, °)

*D*—H⋯*A*	*D*—H	H⋯*A*	*D*⋯*A*	*D*—H⋯*A*
C3—H3⋯O3	0.95	2.32	3.257 (12)	169
C23—H23⋯O3^i^	0.95	2.60	3.453 (12)	150

Symmetry code: (i) 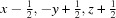.

**Table 2:** Experimental details

Crystal data	
Chemical formula	[Cu(C_16_H_8_Br_3_N_2_O)_2_]·C_2_H_6_OS
*M*_r_	1109.61
Crystal system, space group	Monoclinic, *P*2_1_/*n*
Temperature (K)	150
*a*, *b*, *c* (Å)	8.9922 (14), 16.461 (3), 24.835 (4)
β (°)	92.491 (6)
*V* (Å^3^)	3672.6 (11)
*Z*	4
Radiation type	Mo *K*α
μ (mm^−1^)	7.22
Crystal size (mm)	0.1 × 0.09 × 0.08

Data collection	
Diffractometer	Bruker APEXII
Absorption correction	Multi-scan (*SADABS*; Bruker, 2012 ▸)
*T*_min_, *T*_max_	0.002, 1
No. of measured, independent and observed [*I* > 2σ(*I*)] reflections	6872, 6872, 3962
*R*_int_	0.107
(sin θ/λ)_max_ (Å^−1^)	0.610

Refinement	
*R*[*F* ^2^ > 2σ(*F* ^2^)], *wR*(*F* ^2^), *S*	0.044, 0.099, 0.92
No. of reflections	6872
No. of parameters	446
No. of restraints	150
H-atom treatment	H-atom parameters constrained
Δρ_max_, Δρ_min_ (e Å^−3^)	0.68, −0.50

Computer programs: *APEX2* and *SAINT* (Bruker, 2012 ▸), *SHELXS97* and *SHELXL97* (Sheldrick, 2008 ▸), *ORTEP-3 for Windows* and *WinGX* (Farrugia, 2012 ▸).

## supplementary crystallographic information

### Crystal data

**Table:**

[Cu(C_16_H_8_Br_3_N_2_O)_2_]·C_2_H_6_OS	*F*(000) = 2132
*M**_r_* = 1109.61	*D*_x_ = 1.996 Mg m^−^^3^
Monoclinic, *P*2_1_/*n*	Mo *K*α radiation, λ = 0.7107 Å
Hall symbol: -P 2yn	Cell parameters from 3926 reflections
*a* = 8.9922 (14) Å	θ = 2.6–20.4°
*b* = 16.461 (3) Å	µ = 7.22 mm^−^^1^
*c* = 24.835 (4) Å	*T* = 150 K
β = 92.491 (6)°	Needles, red
*V* = 3672.6 (11) Å^3^	0.1 × 0.09 × 0.08 mm
*Z* = 4	

### Data collection

**Table:**

Bruker APEXII diffractometer	6872 independent reflections
Radiation source: sealed x-ray tube	3962 reflections with *I* > 2σ(*I*)
Graphite monochromator	*R*_int_ = 0.107
φ or ω oscillation scans	θ_max_ = 25.7°, θ_min_ = 1.5°
Absorption correction: multi-scan (SADABS; Bruker, 2012)	*h* = −10→10
*T*_min_ = 0.002, *T*_max_ = 1	*k* = −19→19
6872 measured reflections	*l* = −29→22

### Refinement

**Table:**

Refinement on *F*^2^	Primary atom site location: structure-invariant direct methods
Least-squares matrix: full	Secondary atom site location: difference Fourier map
*R*[*F*^2^ > 2σ(*F*^2^)] = 0.044	Hydrogen site location: mixed
*wR*(*F*^2^) = 0.099	H-atom parameters constrained
*S* = 0.92	*w* = 1/[σ^2^(*F*_o_^2^) + (0.0347*P*)^2^] where *P* = (*F*_o_^2^ + 2*F*_c_^2^)/3
6872 reflections	(Δ/σ)_max_ = 0.001
446 parameters	Δρ_max_ = 0.68 e Å^−^^3^
150 restraints	Δρ_min_ = −0.50 e Å^−^^3^

### Special details

**Table:**

Geometry. All esds (except the esd in the dihedral angle between two l.s. planes) are estimated using the full covariance matrix. The cell esds are taken into account individually in the estimation of esds in distances, angles and torsion angles; correlations between esds in cell parameters are only used when they are defined by crystal symmetry. An approximate (isotropic) treatment of cell esds is used for estimating esds involving l.s. planes.

### Fractional atomic coordinates and isotropic or equivalent isotropic displacement parameters (Å^2^)

**Table:**

	*x*	*y*	*z*	*U*_iso_*/*U*_eq_	Occ. (<1)
Br1	0.68982 (8)	0.64876 (4)	0.67162 (3)	0.0634 (3)	
Br3	0.75391 (7)	0.35743 (4)	0.79468 (3)	0.0584 (3)	
Br2	1.09066 (7)	0.63332 (4)	0.84994 (3)	0.0622 (3)	
Br4	0.21948 (8)	0.19770 (4)	0.78635 (3)	0.0682 (3)	
Br5	−0.21658 (7)	0.22247 (4)	0.61830 (3)	0.0668 (3)	
Br6	0.11254 (7)	0.49832 (4)	0.68081 (3)	0.0556 (3)	
Cu1	0.43783 (7)	0.42742 (4)	0.73579 (3)	0.0398 (2)	
S1A	0.0722 (6)	0.2091 (3)	0.4460 (2)	0.143 (2)	0.700
S1B	0.0869 (10)	0.2867 (7)	0.4489 (3)	0.106 (4)	0.300
O1	0.4041 (4)	0.3614 (2)	0.67460 (16)	0.0503 (16)	
O2	0.4641 (4)	0.4941 (2)	0.79752 (15)	0.0476 (16)	
N1	0.6600 (5)	0.4629 (3)	0.65494 (18)	0.0363 (16)	
N2	0.6228 (4)	0.4666 (3)	0.70433 (18)	0.0361 (16)	
N3	0.2087 (5)	0.3883 (3)	0.81332 (19)	0.0376 (16)	
N4	0.2530 (5)	0.3849 (3)	0.76505 (18)	0.0392 (16)	
C1	0.4550 (6)	0.3729 (3)	0.6277 (2)	0.0405 (19)	
C2	0.3861 (7)	0.3291 (3)	0.5833 (2)	0.0488 (19)	
O3	0.2245 (10)	0.2383 (6)	0.4445 (4)	0.196 (5)	
C3	0.4328 (7)	0.3361 (4)	0.5333 (3)	0.058 (2)	
C4	0.5546 (7)	0.3879 (4)	0.5212 (2)	0.0519 (19)	
C5	0.6008 (9)	0.3970 (5)	0.4671 (3)	0.073 (3)	
C6	0.7130 (9)	0.4461 (5)	0.4561 (3)	0.080 (3)	
C7	0.7873 (8)	0.4909 (5)	0.4968 (3)	0.073 (3)	
C8	0.7460 (7)	0.4844 (4)	0.5492 (3)	0.054 (2)	
C9	0.6276 (6)	0.4321 (3)	0.5625 (2)	0.0429 (17)	
C10	0.5789 (6)	0.4237 (3)	0.6167 (2)	0.0367 (17)	
C11	0.7300 (5)	0.5077 (3)	0.7382 (2)	0.0338 (17)	
C12	0.7737 (6)	0.5880 (3)	0.7296 (2)	0.0389 (17)	
C13	0.8781 (6)	0.6257 (3)	0.7630 (2)	0.0402 (19)	
C14	0.9417 (5)	0.5829 (3)	0.8053 (2)	0.040 (2)	
C15	0.9040 (6)	0.5040 (3)	0.8157 (2)	0.040 (2)	
C16	0.7988 (6)	0.4679 (3)	0.7821 (2)	0.0362 (19)	
C17	0.4038 (6)	0.4840 (3)	0.8433 (2)	0.0396 (17)	
C18	0.4594 (6)	0.5313 (4)	0.8881 (2)	0.052 (2)	
C19	0.4018 (7)	0.5260 (4)	0.9369 (2)	0.058 (2)	
C20	0.2835 (7)	0.4719 (4)	0.9478 (2)	0.0522 (19)	
C21	0.2258 (8)	0.4674 (5)	0.9997 (3)	0.068 (3)	
C22	0.1132 (8)	0.4155 (5)	1.0092 (3)	0.076 (3)	
C23	0.0535 (8)	0.3669 (4)	0.9673 (3)	0.070 (3)	
C24	0.1069 (6)	0.3707 (4)	0.9164 (2)	0.0532 (19)	
C25	0.2235 (6)	0.4237 (3)	0.9057 (2)	0.0410 (17)	
C26	0.2843 (6)	0.4301 (3)	0.8526 (2)	0.0387 (17)	
C27	0.1469 (5)	0.3441 (3)	0.7299 (2)	0.0322 (17)	
C28	0.1148 (6)	0.2620 (3)	0.7347 (2)	0.0407 (19)	
C29	0.0094 (6)	0.2240 (3)	0.7011 (2)	0.0448 (19)	
C30	−0.0631 (6)	0.2696 (3)	0.6626 (2)	0.042 (2)	
C31	−0.0334 (6)	0.3514 (3)	0.6555 (2)	0.042 (2)	
C32	0.0720 (6)	0.3857 (3)	0.6896 (2)	0.0367 (19)	
C33	−0.0284 (15)	0.2589 (10)	0.3984 (5)	0.200 (6)	
C34	−0.0043 (14)	0.2544 (10)	0.4988 (5)	0.200 (6)	
H2	0.30478	0.29405	0.58977	0.0590*	
H3	0.38413	0.30595	0.50504	0.0690*	
H5	0.55076	0.36773	0.43872	0.0870*	
H6	0.74307	0.45080	0.42002	0.0960*	
H7	0.86667	0.52600	0.48811	0.0870*	
H8	0.79692	0.51502	0.57667	0.0640*	
H13	0.90576	0.68054	0.75686	0.0480*	
H15	0.94936	0.47532	0.84525	0.0480*	
H18	0.53963	0.56771	0.88306	0.0620*	
H19	0.44135	0.55961	0.96518	0.0700*	
H21	0.26601	0.50069	1.02799	0.0820*	
H22	0.07469	0.41203	1.04410	0.0910*	
H23	−0.02548	0.33062	0.97419	0.0840*	
H24	0.06465	0.33737	0.88855	0.0640*	
H29	−0.01156	0.16772	0.70477	0.0540*	
H31	−0.08421	0.38228	0.62809	0.0500*	
H33A	−0.12104	0.22926	0.38991	0.3000*	0.700
H33B	0.02909	0.26326	0.36591	0.3000*	0.700
H33C	−0.05193	0.31341	0.41141	0.3000*	0.700
H33D	0.00672	0.20810	0.38277	0.3000*	0.300
H33E	−0.03207	0.30141	0.37076	0.3000*	0.300
H33F	−0.12823	0.25070	0.41182	0.3000*	0.300
H34A	−0.11277	0.24820	0.49574	0.3000*	0.700
H34B	0.02109	0.31232	0.49901	0.3000*	0.700
H34C	0.03409	0.22918	0.53228	0.3000*	0.700
H34D	−0.10002	0.23211	0.48539	0.3000*	0.300
H34E	0.05313	0.21196	0.51788	0.3000*	0.300
H34F	−0.02185	0.29956	0.52342	0.3000*	0.300

### Atomic displacement parameters (Å^2^)

**Table:**

	*U*^11^	*U*^22^	*U*^33^	*U*^12^	*U*^13^	*U*^23^
Br1	0.0881 (5)	0.0411 (4)	0.0592 (4)	0.0021 (3)	−0.0169 (4)	0.0083 (3)
Br3	0.0628 (4)	0.0400 (4)	0.0715 (5)	−0.0095 (3)	−0.0060 (3)	0.0143 (3)
Br2	0.0538 (4)	0.0642 (5)	0.0671 (5)	−0.0118 (3)	−0.0128 (3)	−0.0127 (3)
Br4	0.0725 (5)	0.0558 (4)	0.0742 (5)	0.0048 (4)	−0.0220 (4)	0.0134 (4)
Br5	0.0587 (4)	0.0599 (4)	0.0792 (5)	−0.0140 (3)	−0.0254 (3)	0.0007 (4)
Br6	0.0620 (4)	0.0381 (4)	0.0674 (5)	−0.0064 (3)	0.0115 (3)	0.0044 (3)
Cu1	0.0335 (3)	0.0444 (4)	0.0418 (4)	−0.0075 (3)	0.0062 (3)	−0.0044 (3)
S1A	0.129 (4)	0.094 (3)	0.200 (5)	−0.014 (3)	−0.055 (3)	0.004 (3)
S1B	0.088 (6)	0.135 (8)	0.094 (6)	−0.035 (6)	0.009 (5)	−0.027 (5)
O1	0.048 (2)	0.055 (3)	0.049 (3)	−0.015 (2)	0.015 (2)	−0.013 (2)
O2	0.041 (2)	0.054 (3)	0.048 (3)	−0.0173 (19)	0.0059 (19)	−0.008 (2)
N1	0.035 (2)	0.041 (3)	0.033 (3)	0.002 (2)	0.002 (2)	0.004 (2)
N2	0.033 (2)	0.038 (3)	0.037 (3)	−0.004 (2)	−0.001 (2)	0.000 (2)
N3	0.034 (2)	0.039 (3)	0.040 (3)	0.003 (2)	0.005 (2)	0.003 (2)
N4	0.034 (2)	0.043 (3)	0.041 (3)	−0.009 (2)	0.006 (2)	−0.004 (2)
C1	0.036 (3)	0.038 (3)	0.047 (4)	0.008 (2)	−0.005 (3)	0.000 (3)
C2	0.050 (3)	0.040 (3)	0.055 (4)	0.001 (3)	−0.012 (3)	−0.009 (3)
O3	0.129 (7)	0.242 (10)	0.210 (9)	0.053 (7)	−0.078 (6)	−0.082 (8)
C3	0.068 (4)	0.055 (4)	0.049 (4)	0.011 (3)	−0.013 (3)	−0.014 (3)
C4	0.054 (3)	0.053 (4)	0.048 (3)	0.022 (3)	−0.006 (3)	−0.005 (3)
C5	0.087 (5)	0.086 (5)	0.045 (4)	0.023 (4)	−0.005 (4)	0.000 (4)
C6	0.093 (5)	0.105 (6)	0.043 (4)	0.027 (4)	0.021 (4)	0.015 (4)
C7	0.066 (4)	0.094 (6)	0.059 (4)	0.014 (4)	0.018 (3)	0.027 (4)
C8	0.054 (4)	0.059 (4)	0.048 (4)	0.010 (3)	0.007 (3)	0.015 (3)
C9	0.044 (3)	0.043 (3)	0.042 (3)	0.014 (3)	0.005 (2)	0.000 (3)
C10	0.036 (3)	0.037 (3)	0.037 (3)	0.004 (2)	0.001 (2)	−0.002 (3)
C11	0.037 (3)	0.029 (3)	0.036 (3)	0.000 (2)	0.008 (3)	−0.001 (3)
C12	0.035 (3)	0.039 (3)	0.043 (3)	0.002 (3)	0.005 (3)	−0.001 (3)
C13	0.040 (3)	0.034 (3)	0.047 (4)	−0.002 (3)	0.005 (3)	0.001 (3)
C14	0.030 (3)	0.043 (4)	0.047 (4)	−0.004 (3)	0.002 (3)	−0.011 (3)
C15	0.032 (3)	0.043 (4)	0.045 (4)	−0.001 (3)	0.001 (3)	0.005 (3)
C16	0.035 (3)	0.030 (3)	0.044 (4)	0.000 (2)	0.007 (3)	0.004 (3)
C17	0.034 (3)	0.045 (3)	0.040 (3)	0.002 (2)	0.004 (3)	−0.002 (3)
C18	0.044 (3)	0.061 (4)	0.050 (4)	−0.019 (3)	−0.005 (3)	−0.006 (3)
C19	0.053 (4)	0.080 (5)	0.040 (3)	0.000 (3)	−0.007 (3)	−0.014 (3)
C20	0.044 (3)	0.075 (4)	0.037 (3)	0.010 (3)	−0.004 (3)	0.003 (3)
C21	0.061 (4)	0.106 (6)	0.038 (4)	0.010 (4)	−0.001 (3)	0.000 (4)
C22	0.066 (4)	0.118 (6)	0.045 (4)	0.016 (4)	0.012 (3)	0.012 (4)
C23	0.060 (4)	0.088 (5)	0.063 (4)	−0.001 (4)	0.018 (3)	0.021 (4)
C24	0.051 (3)	0.065 (4)	0.044 (3)	−0.002 (3)	0.006 (3)	0.011 (3)
C25	0.039 (3)	0.050 (3)	0.034 (3)	0.009 (2)	0.002 (2)	0.009 (2)
C26	0.034 (3)	0.042 (3)	0.040 (3)	0.002 (2)	0.000 (2)	0.001 (3)
C27	0.025 (3)	0.035 (3)	0.037 (3)	−0.001 (2)	0.005 (2)	−0.004 (3)
C28	0.040 (3)	0.043 (4)	0.039 (3)	0.000 (3)	0.002 (3)	0.001 (3)
C29	0.041 (3)	0.034 (3)	0.059 (4)	−0.005 (3)	−0.003 (3)	0.005 (3)
C30	0.031 (3)	0.043 (4)	0.050 (4)	−0.005 (3)	−0.003 (3)	−0.001 (3)
C31	0.036 (3)	0.041 (4)	0.049 (4)	0.005 (3)	0.002 (3)	0.006 (3)
C32	0.035 (3)	0.033 (3)	0.043 (4)	−0.006 (2)	0.012 (3)	−0.001 (3)
C33	0.138 (8)	0.360 (16)	0.101 (6)	−0.016 (9)	−0.006 (6)	0.040 (8)
C34	0.138 (8)	0.360 (16)	0.101 (6)	−0.016 (9)	−0.006 (6)	0.040 (8)

### Geometric parameters (Å, º)

**Table:**

Br1—C12	1.884 (5)	C18—C19	1.341 (7)
Br3—C16	1.892 (5)	C19—C20	1.422 (9)
Br2—C14	1.893 (5)	C20—C21	1.412 (9)
Br4—C28	1.883 (5)	C20—C25	1.402 (8)
Br5—C30	1.893 (5)	C21—C22	1.353 (11)
Br6—C32	1.904 (5)	C22—C23	1.401 (10)
Cu1—O1	1.882 (4)	C23—C24	1.373 (9)
Cu1—O2	1.892 (4)	C24—C25	1.398 (8)
Cu1—N2	1.976 (4)	C25—C26	1.453 (7)
Cu1—N4	1.971 (5)	C27—C28	1.388 (7)
S1A—C34	1.681 (14)	C27—C32	1.366 (7)
S1A—O3	1.453 (11)	C28—C29	1.385 (7)
S1A—C33	1.672 (15)	C29—C30	1.360 (7)
S1B—C33	1.657 (16)	C30—C31	1.386 (7)
S1B—C34	1.606 (15)	C31—C32	1.366 (7)
S1B—O3	1.480 (13)	C2—H2	0.9500
O1—C1	1.284 (6)	C3—H3	0.9500
O2—C17	1.292 (6)	C5—H5	0.9500
N1—N2	1.287 (6)	C6—H6	0.9500
N1—C10	1.338 (7)	C7—H7	0.9500
N2—C11	1.423 (6)	C8—H8	0.9500
N3—N4	1.281 (6)	C13—H13	0.9500
N3—C26	1.353 (7)	C15—H15	0.9500
N4—C27	1.432 (7)	C18—H18	0.9500
C1—C2	1.435 (7)	C19—H19	0.9500
C1—C10	1.429 (7)	C21—H21	0.9500
C2—C3	1.333 (9)	C22—H22	0.9500
C3—C4	1.430 (9)	C23—H23	0.9500
C4—C9	1.398 (8)	C24—H24	0.9500
C4—C5	1.431 (9)	C29—H29	0.9500
C5—C6	1.330 (11)	C31—H31	0.9500
C6—C7	1.398 (11)	C33—H33A	0.9800
C7—C8	1.373 (10)	C33—H33B	0.9800
C8—C9	1.419 (8)	C33—H33C	0.9800
C9—C10	1.440 (7)	C33—H33D	0.9800
C11—C12	1.398 (7)	C33—H33E	0.9800
C11—C16	1.394 (7)	C33—H33F	0.9800
C12—C13	1.374 (7)	C34—H34A	0.9800
C13—C14	1.369 (7)	C34—H34B	0.9800
C14—C15	1.370 (7)	C34—H34C	0.9800
C15—C16	1.370 (7)	C34—H34D	0.9800
C17—C26	1.420 (7)	C34—H34E	0.9800
C17—C18	1.430 (7)	C34—H34F	0.9800
			
O1—Cu1—O2	177.90 (16)	N3—C26—C25	114.9 (5)
O1—Cu1—N2	88.75 (18)	N4—C27—C32	120.4 (5)
O1—Cu1—N4	89.07 (18)	C28—C27—C32	117.0 (5)
O2—Cu1—N2	93.06 (17)	N4—C27—C28	122.7 (4)
O2—Cu1—N4	89.13 (18)	C27—C28—C29	121.8 (5)
N2—Cu1—N4	177.8 (2)	Br4—C28—C27	120.4 (4)
C33—S1A—C34	96.1 (7)	Br4—C28—C29	117.7 (4)
O3—S1A—C33	107.5 (7)	C28—C29—C30	118.0 (5)
O3—S1A—C34	106.9 (7)	Br5—C30—C29	119.8 (4)
O3—S1B—C34	109.6 (9)	Br5—C30—C31	117.6 (4)
O3—S1B—C33	107.1 (9)	C29—C30—C31	122.5 (5)
C33—S1B—C34	99.7 (8)	C30—C31—C32	117.1 (5)
Cu1—O1—C1	126.6 (3)	C27—C32—C31	123.6 (5)
Cu1—O2—C17	126.6 (3)	Br6—C32—C27	118.8 (4)
N2—N1—C10	122.9 (5)	Br6—C32—C31	117.6 (4)
Cu1—N2—C11	118.8 (3)	C1—C2—H2	119.00
Cu1—N2—N1	128.3 (3)	C3—C2—H2	119.00
N1—N2—C11	112.9 (4)	C2—C3—H3	119.00
N4—N3—C26	122.0 (5)	C4—C3—H3	119.00
Cu1—N4—N3	129.1 (4)	C6—C5—H5	120.00
Cu1—N4—C27	119.5 (3)	C4—C5—H5	120.00
N3—N4—C27	111.4 (4)	C5—C6—H6	120.00
O1—C1—C10	124.9 (5)	C7—C6—H6	119.00
O1—C1—C2	117.6 (5)	C8—C7—H7	120.00
C2—C1—C10	117.5 (5)	C6—C7—H7	120.00
C1—C2—C3	122.0 (5)	C7—C8—H8	120.00
C2—C3—C4	121.5 (6)	C9—C8—H8	120.00
C5—C4—C9	119.2 (6)	C12—C13—H13	121.00
C3—C4—C9	119.7 (5)	C14—C13—H13	121.00
C3—C4—C5	121.1 (6)	C16—C15—H15	121.00
C4—C5—C6	120.7 (7)	C14—C15—H15	121.00
C5—C6—C7	121.0 (7)	C17—C18—H18	119.00
C6—C7—C8	120.3 (7)	C19—C18—H18	119.00
C7—C8—C9	120.3 (6)	C18—C19—H19	119.00
C4—C9—C10	118.9 (5)	C20—C19—H19	119.00
C4—C9—C8	118.6 (5)	C22—C21—H21	120.00
C8—C9—C10	122.5 (5)	C20—C21—H21	120.00
C1—C10—C9	120.4 (5)	C21—C22—H22	120.00
N1—C10—C1	123.5 (5)	C23—C22—H22	120.00
N1—C10—C9	116.0 (5)	C22—C23—H23	119.00
N2—C11—C16	120.4 (4)	C24—C23—H23	119.00
C12—C11—C16	116.5 (4)	C25—C24—H24	120.00
N2—C11—C12	123.2 (5)	C23—C24—H24	120.00
C11—C12—C13	121.6 (5)	C28—C29—H29	121.00
Br1—C12—C11	120.9 (4)	C30—C29—H29	121.00
Br1—C12—C13	117.5 (4)	C32—C31—H31	121.00
C12—C13—C14	118.9 (5)	C30—C31—H31	121.00
Br2—C14—C13	119.0 (4)	S1A—C33—H33A	110.00
Br2—C14—C15	118.7 (4)	S1A—C33—H33B	109.00
C13—C14—C15	122.3 (5)	S1A—C33—H33C	109.00
C14—C15—C16	117.8 (5)	S1B—C33—H33D	109.00
C11—C16—C15	123.0 (5)	S1B—C33—H33E	110.00
Br3—C16—C11	119.4 (4)	S1B—C33—H33F	109.00
Br3—C16—C15	117.6 (4)	H33A—C33—H33B	109.00
O2—C17—C18	118.0 (5)	H33A—C33—H33C	109.00
O2—C17—C26	125.0 (5)	H33B—C33—H33C	110.00
C18—C17—C26	117.1 (5)	H33D—C33—H33E	109.00
C17—C18—C19	122.1 (5)	H33D—C33—H33F	109.00
C18—C19—C20	122.3 (5)	H33E—C33—H33F	109.00
C19—C20—C25	118.6 (5)	S1A—C34—H34A	109.00
C19—C20—C21	120.9 (6)	S1A—C34—H34B	109.00
C21—C20—C25	120.4 (6)	S1A—C34—H34C	109.00
C20—C21—C22	120.0 (7)	S1B—C34—H34D	109.00
C21—C22—C23	119.8 (7)	S1B—C34—H34E	109.00
C22—C23—C24	121.3 (6)	S1B—C34—H34F	109.00
C23—C24—C25	119.9 (5)	H34A—C34—H34B	109.00
C20—C25—C26	119.2 (5)	H34A—C34—H34C	110.00
C20—C25—C24	118.6 (5)	H34B—C34—H34C	110.00
C24—C25—C26	122.2 (5)	H34D—C34—H34E	110.00
C17—C26—C25	120.7 (4)	H34D—C34—H34F	109.00
N3—C26—C17	124.1 (5)	H34E—C34—H34F	110.00
			
N2—Cu1—O1—C1	−24.1 (4)	N2—C11—C12—Br1	0.7 (7)
N4—Cu1—O1—C1	156.5 (4)	N2—C11—C12—C13	−179.7 (5)
N2—Cu1—O2—C17	−159.5 (4)	C16—C11—C12—Br1	179.9 (4)
N4—Cu1—O2—C17	20.0 (4)	C16—C11—C12—C13	−0.5 (7)
O1—Cu1—N2—N1	18.6 (5)	N2—C11—C16—Br3	2.4 (6)
O1—Cu1—N2—C11	−164.4 (4)	N2—C11—C16—C15	179.2 (5)
O2—Cu1—N2—N1	−160.4 (5)	C12—C11—C16—Br3	−176.9 (4)
O2—Cu1—N2—C11	16.7 (4)	C12—C11—C16—C15	0.0 (8)
O1—Cu1—N4—N3	164.0 (5)	Br1—C12—C13—C14	−179.6 (4)
O1—Cu1—N4—C27	−16.5 (4)	C11—C12—C13—C14	0.8 (8)
O2—Cu1—N4—N3	−17.1 (5)	C12—C13—C14—Br2	177.8 (4)
O2—Cu1—N4—C27	162.4 (4)	C12—C13—C14—C15	−0.5 (8)
Cu1—O1—C1—C2	−163.7 (4)	Br2—C14—C15—C16	−178.3 (4)
Cu1—O1—C1—C10	18.8 (7)	C13—C14—C15—C16	0.0 (8)
Cu1—O2—C17—C18	168.1 (4)	C14—C15—C16—Br3	177.2 (4)
Cu1—O2—C17—C26	−12.9 (7)	C14—C15—C16—C11	0.3 (8)
C10—N1—N2—Cu1	−6.0 (8)	O2—C17—C18—C19	178.7 (5)
C10—N1—N2—C11	176.8 (5)	C26—C17—C18—C19	−0.4 (8)
N2—N1—C10—C1	−8.9 (8)	O2—C17—C26—N3	−6.8 (8)
N2—N1—C10—C9	174.0 (5)	O2—C17—C26—C25	−179.5 (5)
Cu1—N2—C11—C12	−118.7 (5)	C18—C17—C26—N3	172.2 (5)
Cu1—N2—C11—C16	62.1 (6)	C18—C17—C26—C25	−0.5 (7)
N1—N2—C11—C12	58.8 (6)	C17—C18—C19—C20	1.3 (10)
N1—N2—C11—C16	−120.4 (5)	C18—C19—C20—C21	179.6 (6)
C26—N3—N4—Cu1	5.4 (8)	C18—C19—C20—C25	−1.2 (9)
C26—N3—N4—C27	−174.2 (5)	C19—C20—C21—C22	180.0 (7)
N4—N3—C26—C17	10.4 (8)	C25—C20—C21—C22	0.9 (11)
N4—N3—C26—C25	−176.5 (5)	C19—C20—C25—C24	−179.8 (6)
Cu1—N4—C27—C28	113.6 (5)	C19—C20—C25—C26	0.4 (8)
Cu1—N4—C27—C32	−66.5 (6)	C21—C20—C25—C24	−0.7 (9)
N3—N4—C27—C28	−66.8 (6)	C21—C20—C25—C26	179.5 (6)
N3—N4—C27—C32	113.2 (5)	C20—C21—C22—C23	−0.5 (11)
O1—C1—C2—C3	−178.9 (5)	C21—C22—C23—C24	0.0 (11)
C10—C1—C2—C3	−1.3 (8)	C22—C23—C24—C25	0.2 (10)
O1—C1—C10—N1	2.7 (8)	C23—C24—C25—C20	0.1 (9)
O1—C1—C10—C9	179.7 (5)	C23—C24—C25—C26	180.0 (6)
C2—C1—C10—N1	−174.8 (5)	C20—C25—C26—N3	−172.9 (5)
C2—C1—C10—C9	2.2 (7)	C20—C25—C26—C17	0.5 (8)
C1—C2—C3—C4	0.0 (9)	C24—C25—C26—N3	7.3 (8)
C2—C3—C4—C5	−178.0 (6)	C24—C25—C26—C17	−179.4 (5)
C2—C3—C4—C9	0.4 (10)	N4—C27—C28—Br4	−3.5 (7)
C3—C4—C5—C6	179.2 (7)	N4—C27—C28—C29	178.5 (5)
C9—C4—C5—C6	0.8 (11)	C32—C27—C28—Br4	176.5 (4)
C3—C4—C9—C8	−178.6 (6)	C32—C27—C28—C29	−1.4 (8)
C3—C4—C9—C10	0.6 (8)	N4—C27—C32—Br6	0.5 (7)
C5—C4—C9—C8	−0.3 (9)	N4—C27—C32—C31	−178.2 (5)
C5—C4—C9—C10	179.0 (6)	C28—C27—C32—Br6	−179.6 (4)
C4—C5—C6—C7	−0.9 (12)	C28—C27—C32—C31	1.7 (8)
C5—C6—C7—C8	0.5 (12)	Br4—C28—C29—C30	−177.9 (4)
C6—C7—C8—C9	0.0 (11)	C27—C28—C29—C30	0.1 (8)
C7—C8—C9—C4	−0.1 (9)	C28—C29—C30—Br5	−176.3 (4)
C7—C8—C9—C10	−179.3 (6)	C28—C29—C30—C31	1.1 (8)
C4—C9—C10—N1	175.3 (5)	Br5—C30—C31—C32	176.6 (4)
C4—C9—C10—C1	−1.9 (8)	C29—C30—C31—C32	−0.8 (8)
C8—C9—C10—N1	−5.5 (8)	C30—C31—C32—Br6	−179.4 (4)
C8—C9—C10—C1	177.3 (5)	C30—C31—C32—C27	−0.7 (8)

### Hydrogen-bond geometry (Å, º)

**Table:**

*D*—H···*A*	*D*—H	H···*A*	*D*···*A*	*D*—H···*A*
C3—H3···O3	0.95	2.32	3.257 (12)	169
C23—H23···O3^i^	0.95	2.60	3.453 (12)	150

Symmetry code: (i) *x*−1/2, −*y*+1/2, *z*+1/2.
